# Men’s involvement in family planning programs: an exploratory study from Karachi, Pakistan

**DOI:** 10.1186/s12978-024-01875-1

**Published:** 2024-10-03

**Authors:** Jawaria Mukhtar Ahmed, Farina Gul Abrejo, Xaher Gul, Sarah Saleem

**Affiliations:** 1 Aga Khan University, Karachi, Pakistan; 2 Visiting Faculty AKU, Aga Khan University, Karachi, Pakistan; 3 Population and Reproductive Health Section of CHS, AKU, Aga Khan University, Karachi, Pakistan

**Keywords:** Family planning, Men involvement, Reproductive health, Low- and middle-income

## Abstract

**Background:**

In Pakistan, family planning has traditionally been perceived as primarily a women’s concern, resulting in the exclusion of men from relevant initiatives. This situation is further exacerbated by cultural and social barriers that hinder men’s access to family planning services. This study addresses a significant research gap by assessing the extent of family planning service provision for men in urban areas of Karachi. It delves into the exploration of men’s involvement in family planning service delivery, identifies existing gaps in services catering to men, records men’s perceptions of accessibility and acceptability of these services, and ultimately offers recommendations to enhance men’s involvement and strengthen service provision to better meet their needs.

**Methods:**

We employed a qualitative exploratory research design using semi-structured interviews to investigate perceptions regarding family planning service provision to men. This approach involved 25 interviews, comprising eight key informant interviews with stakeholders, eight with service providers, and nine in-depth interviews with married men.

**Results:**

This study highlights the limited engagement of men in family planning programs, primarily due to entrenched sociocultural norms that confine female healthcare providers to serving women, hindering men’s involvement. While national and provincial policies endorse men’s participation, they lack defined roles for male providers. Behavioral and information-sharing barriers at the community level discourage male healthcare providers from collaborating with females. Family planning programs, except for NGO-led vasectomy projects, fail to adequately address men’s needs. Despite policy recognition, implementation remains inadequate. Bridging the men’s involvement gap necessitates more male providers and improved contraceptive stigma combat training. Further research is vital to explore effective methods for involving men in community and service delivery in family planning.

**Conclusion:**

There is a need to change the perception that contraception is solely the responsibility of women, as men’s participation in family planning in Karachi is limited. Engaging men can yield positive health and non-health outcomes. Culturally sensitive services, developed with community input using a couple-centered approach, are crucial for equitable family planning. Further research is needed to explore men’s inclusion strategies in service provision and utilization.

## Abstract (plain language summary)

Many family planning efforts focus solely on women, leaving out men, especially in Pakistan, due to cultural and social barriers. This study aimed to explore men’s involvement in family planning programs in Karachi. We talked to 25 different people, such as those who run family planning programs, provide services, and married men. We found that men aren’t much involved in these programs. Female health providers usually help women, which discourages men from participating or getting involved in such programs. Even though there have been policies to include men since 2002, they don’t have specific roles defined. Men don’t  discuss family planning much with female providers due to behavioral differences and lack of open communication. Men mostly prefer condoms, and apart from a small NGO-run vasectomy project, there are few programs-tailored to their needs. Although policies exist, they’re not fully in practice. To change this, we need more male providers and improved training to reduce the stigma around contraception. Future research should  explore more effective ways to involve men in family planning, both in communities and service levels. Overall, getting men involved in family planning is important, and services need to be developed with input from communities and couples to ensure fairness. More research is needed to figure out the best ways to do this.

## Background

The approach to family planning provisions that prioritize human rights, involves giving men and women equal opportunities to make decisions about the number and timing of their children. The International Conference on Population and Development (ICPD) has supported this approach, encouraging equal access to family planning information, products, and services for both men and women [1]. International organizations and global conferences have further reinforced this rights-based principle [2]. Recent agendas for family planning, such as FP 2030 and sustainable developmental goals (SDGs), also reinforce the importance of men’s involvement in family planning programs and work towards fostering a supportive environment that engages men as partners in reproductive health decision-making. By building on each other’s principles and commitments, they emphasize creating more inclusive and effective family planning programs that involve and support men as partners in reproductive health decisions. High-income countries have already adopted a couple-centered approach to promote family planning services by involving men in reproductive health decision-making [3–5], and many low and middle-income countries (LMICs) are also working to promote equality in family planning by revising or implementing family planning programs and services that involve men [6]. For example, Nigeria, Malawi, and Ethiopia are exploring ways to understand men’s perceptions and attitudes regarding family use [7–9]. Other countries include men as providers of family planning services to fulfill men’s need for family planning services at the community level. Efforts are also being made to remodel community health programs and policies to ensure men’s involvement in family planning. However, cultural, and social segregation continues to pose obstacles [10–12].

In Bangladesh, a single, limited initiative aimed at educating men about reproductive health was carried out in 2000. In order to provide male reproductive health services in rural Health and Family Welfare Centers (HFWCs), which have historically focused on women’s reproductive health issues, the project trained 127 service providers and field workers. Religious scholars have a significant impact on public opinion and behavior in Bangladesh. The Islamic Foundation, in partnership with the Ministry of Health and Family Planning and the Family Planning Association of Bangladesh, has started discussions with these leaders in order to promote family planning and male responsibility in this regard. These initiatives seek to clarify misconceptions and promote a family planning-friendly view based on the Quran and Hadith. Over 40,000 imams had received training from UNFPA and the Ministry of Religious Affairs by 2006; assessments reveal that at least 40% of them bring up family planning in social gatherings and Jumma prayers [13].

As a signatory of international agendas, including FP2030, Pakistan acknowledges the significance of engaging men in family planning programs [14]. Family planning, along with health, are both provincial subjects post-devolution under the 18th amendment and the Government of Sindh is also a signatory to FP 2030 commitments [15].

To accomplish the 2030 family planning (FP) targets, the following goals were set for Sindh province based on the Pakistan Demographic and Health Survey (PDHS) report 2017–2018.

i. Increase the contraception prevalence rate (CPR) to 43% by 2025 and 50% by 2030.
ii. Reduce the total fertility rate (TFR) to 3.0% by 2025 and 2.6% by 2030.
iii. Decrease Annual population growth rate to 1.5% by 2025 and 1.3% by 2030 [16].

The Sindh Population Welfare Department (PWD) owns the primary mandate for delivering family planning services in Sindh, supported by the Department of Health (DoH). Both departments fall under the Ministry of Health and Population Welfare and provide family planning services through public sector facilities across Sindh province, including via public-private partnerships.

Limited male involvement in reproductive health and family planning initiatives in Pakistan has persisted despite well-intentioned efforts, such as the introduction of social male mobilizers working alongside community health workers. These initiatives aim to raise awareness about fatherhood, catalyze social change, and guide men within communities toward embracing their roles and responsibilities [17]. Initiatives to engage men in family planning face challenges. These challenges include deeply entrenched cultural norms, men’s lack of awareness about their role, and stigma around contraception. This, in turn, leads to increased unintended pregnancies, sexually transmitted infections, and financial strain on families. The dearth of male-friendly family planning services, coupled with the broader weaknesses in healthcare infrastructure, including the shortage of trained personnel and inconsistent contraceptive supply, also contribute to this. Moreover, the absence of carefully tailored strategies to engage men in family planning efforts, alongside a lack of supportive policies and guidelines to integrate meaningful involvement of men, presents a significant stumbling block [18].

Resistance from various stakeholders, including healthcare providers, community leaders, and even men themselves, further obstructs the shift in attitudes and practices necessary to enhance men’s participation in family planning [19]. Another pivotal factor is the historical framing of family planning as predominantly a female concern, leading to the disproportionate targeting of women in family planning programs. Insufficient emphasis has been placed on crafting strategies to actively involve men and cultivate their engagement in reproductive health initiatives. This unbalanced approach failed to reflect the evolving needs of both men and women resulting in stagnant contraceptive prevalence over the past decade. Urgent attention is needed to reshape the narratives around family planning and create a more inclusive and comprehensive approach that acknowledges the crucial role men play in family planning and reproductive health. This study addresses a gap in research on men’s involvement in family planning programs in Karachi, Pakistan, and aims to explore the extent of men’s engagement, identify barriers and gaps in current programs, and suggest strategies for improved men’s participation. Examining men’s perspectives, knowledge, and challenges contributes to understanding how they perceive their role in family planning, informing interventions for gender equity and reproductive health improvements. Such insights are critical for policymakers, healthcare providers, and researchers working in LMICs and South Asia, where involving men can address unmet needs and promote gender equality.

## Methods

We conducted an exploratory, descriptive, qualitative approach to gain an understanding of the health systems and their ability to offer services to men within communities. This study design allows researchers to study individuals in-depth and collect enriched data. It involves inquiring questions and probing participants’ responses to get a range of thoughts and evolving themes. This approach is suitable for this study as it helped the researcher to collect enriched data with a significant amount of information from small focus subject groups. The exploratory qualitative study provided crucial insights into gaps and challenges faced by males and service providers while using or giving services specific to male contraceptives. It also helped us know how our health system addresses male needs and engages in the family planning program. Purposive sampling was done to identify the stakeholders. Participants were recruited based on gender, the service providers’ positions, and their work departments. Stakeholders from the Population Welfare Department (PWD), Ministry of Health (MoH), Managers, and Directors from different NGOs and organizations working for family planning services were included having experience of a minimum of three years in the same position. Service providers from the public and private sectors working in hospital-based setups, NGO-based setups, and semi-private setups were included to understand the nature of services available, their accessibility to males, and their experience in providing contraceptives, with a minimum of nine months of experience. Married Males from different areas of Karachi were recruited for this study. Males who have been married for at least the last two years and are residents of Karachi were selected for this study.

Eligibility Criteria:

### Inclusion criteria for study participants

- Males have been residing in different parts of Karachi for at least last year.
- Males of 20–50 years of age.
- Males who have lived with their spouses for the last six months.
- Males planning to have children or at least have one child.
- Service providers have at least nine months of experience in FP services provision.
- Stakeholders have at least three years of working experience in that position.

### Exclusion criteria for study participants

- Travelers or tourists visiting Karachi.
- Any male who is not falling above the stated age limit.
- Males who have not been living with their spouses for the last six months are due for any reason.
- Service providers have less than nine months of experience in FP service provision.

This study utilized in-depth and key informant interviews to understand participants’ perceptions of the provision and reception of family planning services, as well as perspectives of service providers, policymakers, and married men on the services directed towards men. We developed three semi-structured interview guides. The key informant interviews were conducted with a group of eight stakeholders, including directors, high-level managers, and program coordinators employed in the province’s public and private sectors of family planning programs in Sindh province. The second group of key informant interviews included eight family planning service providers, including Lady Health Workers, Community Health Workers, Male Social Mobilizers, and medical doctors. These providers serve various sectors and areas in Karachi. In addition, in-depth interviews were conducted with nine married men. Out of nine married men two had completed their education up to the intermediate level, two had bachelor’s degrees, and five had achieved a master’s level education and were currently residing with their spouses. All interviews were conducted until the point of saturation. The interview guidelines were prepared in the English language and translated into the Urdu language. A pilot test was conducted to detect any changes in the meaning of the content lost during translations. Bilingual experts were intricately involved to ensure precision, employing meticulous steps such as back translation and expert reviews. The interviews were transcribed verbatim in the Urdu language and then translated into the English language for analysis. These bilingual insights and adjustments refined translations, guaranteeing a high level of accuracy and fidelity to the original content. Our team comprised a social mobilizer, a bilingual expert, a note-taker, a social behavior change expert, and a research fellow with extensive experience in qualitative research. The interviews were conducted from May to September 2022, and the study was approved by the Ethical Review Committee of the Aga Khan University, Karachi (ERC # 2022-7136-21536).

### Conceptual framework of the study

This study used the World Health Organization (WHO) Health System Building Blocks as the guiding framework to understand men’s involvement in family planning for both the provision and utilization of services. In multiple ways, the six building blocks support the improvement of health systems. The foundation for general policy and regulation of all the other building blocks of the health system is provided by several cross-cutting elements, such as leadership/governance and health information systems. Finance and the health workforce are two examples of key input components for the healthcare system. A third category includes the delivery of medical services and goods, representing how the health system ensures accessibility and distribution of treatment [20]. This framework allows for the establishment of indicators and measurement methodologies for tracking progress toward the involvement of men and helps focus on these distinct elements and set limits around this complicated construct.

**Fig. 1:**
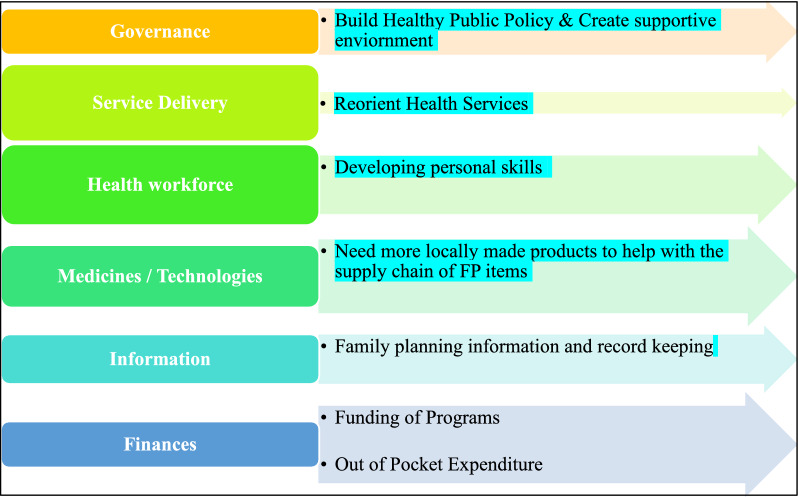
Illustrating the pre-defined themes and Sub-themes of the data

In this study, a deductive approach was used to describe findings within each of the six building blocks as major themes. Further, the subthemes of this study were aligned with the five pillars of the Ottawa Charter for Health Promotion framework. This paradigm aided the researcher in thoroughly examining stakeholders', service providers', and men beneficiaries’ perceptions of men’s participation in family planning programs. The governance and information blocks provided limited information in this study. The researcher modifies the framework to emphasize the importance of men’s involvement in family planning programs. Figure 1 defines a conceptual framework developed around those blocks. This helps in identifying patterns, responses, or meanings within these themes [21].

### Study procedure

Before conducting interviews, we provide participants with a brief overview of the study and assured them of confidentiality. Written informed consent was obtained from each participant. The study ensured informed consent and confidentiality for all participants, which are crucial ethical practices. Additionally, it’s essential to highlight that the research strictly followed established ethical guidelines, including those specified in the Declaration of Helsinki, to safeguard the well-being and rights of all involved human subjects. Interviews were conducted in private settings, and participants explicitly consented to audio recording, which were later transcribed and translated. The duration of each interview ranged from 30 to 45 min. We enrolled stakeholders who had been working at the managerial level or at director level for at least three years in both the public and private sector. For the service providers, permission was taken from the District Health Officer (RHMNCH) Karachi East. The service providers were recruited from the public, private, and PPHI. In the initial phase all the stakeholders and service providers were physically approached to obtain consent and their availability for an interview. Upon receiving verbal consent, written consent was obtained before each interview.

### Data Analysis

**Fig. 2:**
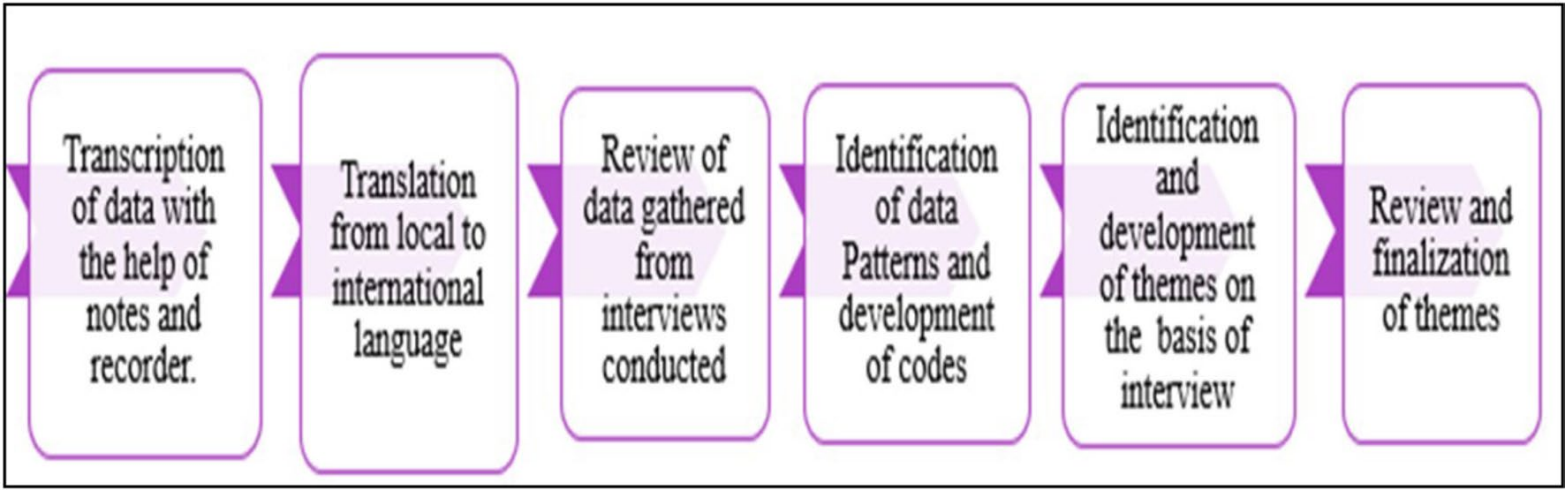
Steps of thematic analysis

The data were analyzed using NVivo 15, using predefined themes. The analysis followed a deductive approach (top-down approach) where the researcher began with a broad hypothesis and then evaluated it using detailed observations. The study’s data analysis and interpretation relied on participant narratives, cross-checked with recordings. The analysis involved steps from transcribing all interviews on NVivo to coding text into themes, merging them into categories, and drawing inferences for analysis. The results were backed by direct quotes from interviews to authenticate findings and were overseen by the researcher and the co-researchers for confirmation. When a researcher has a specific study question, this strategy performs well [22]. The deductive approach helps to identify the patterns, responses, and experiences of participants related to the phenomenon. This involved organizing and categorizing the data, aligning it with the research question, and utilizing a theoretical framework to develop codes for sorting the data into predetermined categories [23]. The process described in Fig. 2 below was used to analyze the data.

The trustworthiness of the data was maintained using four criteria.

**Fig. 3:**
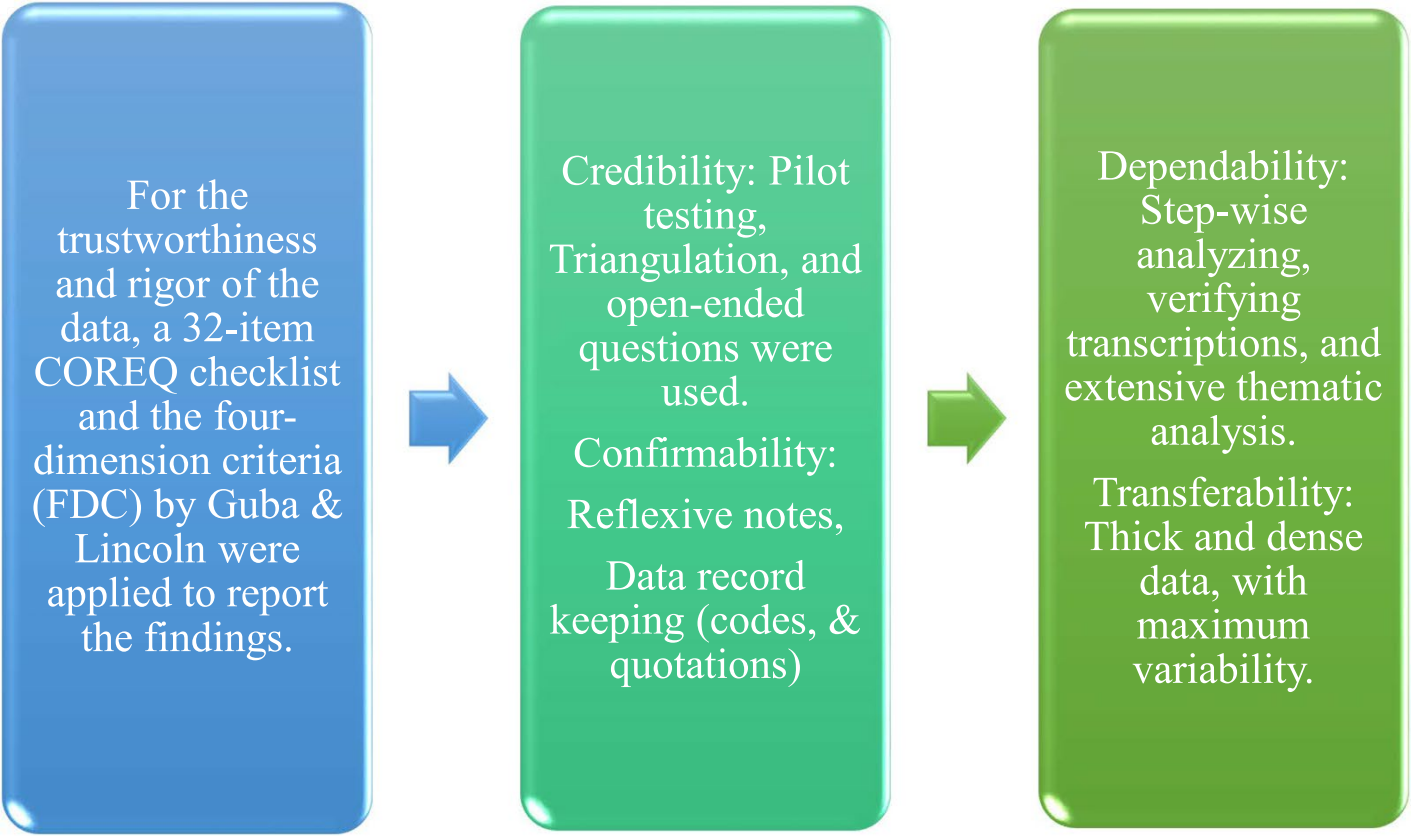
Trustworthiness and Rigor of the Study

The findings were reported using 32 items listed in the COREQ checklist [23]. The study was triangulated using one out of four types of triangulations suggested by Denzin [24]; data triangulation. Figure 3 below comprehensively shows the trustworthiness and rigor of the study.

The trustworthiness of the study was ensured through prolonged engagement with participants, member checking, data triangulation, peer debriefing, and meticulous documentation of the research process. The results of the study were also ensured by using these methods. The study used prolonged engagement with participants to ensure that their voices were accurately represented. This means that the researchers spent a lot of time with the participants, getting to know them and their experiences. They also used member checking, which is a process of sharing the findings of the study with the participants to get their feedback. This helps to ensure that the findings are accurate and that the participants' voices were heard.

Data triangulation was used to strengthen the credibility of the study. This means that the researchers used multiple respondents to collect data. This helps to ensure that the findings were consistent and that they could be corroborated from multiple sources. Transferability was addressed by providing a detailed description of the study context and participants. This means that the researchers provided enough information about the study so that the findings could be applied to other settings.

Dependability was ensured by engaging in peer debriefing throughout the research process. This means that the researchers sought feedback from colleagues or experts on the methods, findings, and interpretations of the study. This helped to ensure that the study was conducted in a rigorous and unbiased manner. Conformability was achieved through peer debriefing and an audit trail of decision-making. This means that the researchers documented their decisions throughout the research process. This helped to ensure that the study was transparent and that the findings could be replicated by other researchers.

Overall, the study was conducted in a rigorous and trustworthy manner. The researchers use a variety of methods to ensure that the findings are accurate, credible, and transferable to different similar LMICs contexts. Guaranteeing research trustworthiness faces limitations, including securing credible data collection of sensitive information, overcoming logistical and cultural obstacles for participant engagement, and upholding data reliability amidst biases or measurement errors. The data analysis may be restricted by limited tools or expertise, while contextual interpretation of findings can be challenging without diverse viewpoints. Effectively communicating findings to stakeholders of varying expertise levels poses an additional hurdle. Addressing these demands necessitates meticulous planning, diverse methodologies, and unwavering adherence to transparency and reflexivity throughout the research endeavor.

### Reflexivity

Each team member contributed their unique perspectives and experiences to the observation process, ensuring a comprehensive understanding of the various scenarios encountered during the interviews. Their diverse expertise allowed them to identify and interpret subtle cues and nuances that might have been overlooked by a single individual. This collaborative approach enhances the credibility and reliability of the observations, ensuring that the data gathered accurately reflects the complexities of the interview settings.

## Results

The demographic characteristics of the participants (Table 1) show that most stakeholders are men and belong to the private sector.

**Table:** 

Variables	Stakeholders	Service providers	Married men
Age (Mean age in years)	39.1	44.7	36.7
Gender Female Male	37.5% 62.5%	62.5% 37.5	- 100%
Public Sector	8%	16%	-
Private Sector	24%	12%	-
Semi-Private	-	4%	-

Predetermined themes and subthemes were utilized to examine the data. These main themes included the service delivery, governance, health workforce, medicine/technologies, finances, and information. Further details of each theme and sub-theme are provided below:

### Service delivery

#### Reorient health services

The theme of men’s engagement in service provision and usage is significant and includes key components, such as men’s participation in service delivery, their accessibility and availability to services, and the existing family planning services aimed at meeting men’s needs. The identified sub-themes were as follows:

Involving men in service delivery is an essential component of the family planning program and was explored during stakeholder interviews. *The cultural and societal aspects* were dominant when the stakeholders and providers responded to this question, where it was emphasized that:

*“men’s involvement in the family planning services provision is not culturally accepted in our society.”*

However, there is a cadre of male mobilizers working in the public sector parallel to lady health workers in many parts of the province, but their impact is yet to be observed. As one of the SH_002 mentioned,

*"Changing behavior is the responsibility of male mobilizers*,* but they are significantly rejected by male members of society; they are abused*,* humiliated*,* and beaten up by men in the communities”*. “*Lack of acceptability in the communities for these male mobilizers"*.

is one of the reasons discussed during the interviews by SH_004. *The geographical context* was also considered one of the factors associated with cultural acceptance to involve men in family planning service provision. As one of the participants SH_005 mentioned,

*“In urban areas*,* male providers can sit and counsel couples with a female provider*,* but in semi-urban areas it is difficult for men to visit communities and talk to male members for family planning there”.*

The mechanism adopted by the communities to involve men was also explored through interviews with stakeholders and service providers. *“Mohalla (communities) meetings”* was the only mechanism that was highlighted during these interviews. These meetings were conducted once or twice a month by male mobilizers; however, very few men from the communities attended these meetings. One of the SP_009 said:

"*We conduct mohalla meeting twice a month in which 6–7 men come".*

Accessibility of male mobilizers or male providers to RHC and BHU clinics was also explored, where it was discussed that these providers never visited these clinics. While inquiring about the accessibility of the programs and availability of door-to-door services one of the MM_003 commented,

“*No*,* these programs are not easily available in the community they are very less*”.

While MM_004 said,

“*No*,* they’re not. I have never met any person at my doorstep telling me about reproductive health or informing me about family planning or things of those sorts. So*,* I have not met them in my 30 years of life*”.

All participants were asked about service availability and accessibility for men living in the communities. The stakeholders unanimously mentioned that there was no specific family planning program for changing behavior or addressing men’s need for family planning in the region (specifically Karachi). While SH_005 commented,

“*…I have never heard of or seen any program focusing on men’s family planning needs”*.

The major argument against this gap is due to the limited availability of male contraceptive methods; SH_007 said,

“*Offering men-oriented project would be a waste of time…as there is nothing to tell men to accept vasectomy or condoms in Pakistan”.*

A female SP_003 also discussed,

*We do not talk to men. There are no specific services available for them.*

The shortage of male doctors was also considered one of the reasons for these limited offerings; it was discussed that,

“*Vasectomy is only available in those areas where there are male doctors*,* so if we talk to men about this method*,* we are not sure where they would avail those services”* (SP_005).

SP_003 considered that,

*“They needed to involve men if they required consent”.*

On the other hand, men from the communities were asked if they knew of any men’s contraceptive services provided at facilities such as RHC or BHU in Karachi. Most participants did not know about family planning facilities. Still, they knew about a few social marketing products such as “*green star”* (a private organization), or *“Khandani mansooba bandi* (family planning) *program”* Khandani mansooba bandi family planning program (public program), as these programs are advertised on media. However, they were questioned about whether anyone approached them in the communities for these services. Most of them mentioned that no one had ever visited them to offer these services. One MM_005 said,

“*I have never been visited by any of the workers who visited my home to discuss the family planning program with me”.*

While responding to the question about men’s visiting family planning programs, one participant MM_009 said,

“*Men don’t go to any such clinics*,* but those who have some infertility issues or have no children…men don’t go anywhere to prevent unwanted children”.*

Another participant MM_007 specifically mentioned,

*“I’ve only heard of women going to those clinics; I’ve never known any man to go there.”*

Family planning services aim to bridge gender gaps by addressing the specific needs of men in communities. These programs and services are designed to understand and meet individual needs effectively. Stakeholders responded that there are currently no such initiatives. The government is planning to introduce programs to change men’s behavior towards family planning use, but currently, it is difficult to involve them as only female workers are providing services in the communities. An SH_004 discussed,

“*It is difficult for female workers to talk to men and discuss family planning with men in the communities. We have a patriarchal society*,* and it is culturally inappropriate to involve men in such programs”*.

This argument was also affirmed by a female family SP_002 who said,

*We cannot involve men or change their behavior because we do not interact with them”.* Another SP_006 said they “*indirectly involve men as they discuss these things with their wives*,* and we use them to convey family planning messages to men.*

Similarly, all male participants said that,

*“They were unaware of a program focusing on behavior change or providing male contraception directly to them”.*

### Medicine/technologies

#### Need more locally made products to help with the supply chain of FP items

The theme was explored to understand the availability of family planning commodities to fulfill the needs of men living in communities.

According to stakeholders, the shortage of commodities was attributed to the import of family planning commodities as a major cause. During the COVID pandemic, there has been an increase in the shortage of family planning commodities leading to increased unintended pregnancies, increased spread of sexually transmitted infections (STIs), decreased access to abortion services, and reduced access to reproductive healthcare. The SH_002 mentioned,

“*The shortage at the post-covid*,* and during covid was high*,* and demand was difficult to meet during this time”*.

Providers, on the other hand, affirm the shortage; a provider from the private sector SP_004 said,

*We consistently encounter the issue of stock-outs every quarter*.

A female SP-005 mentioned,

“*Six to eight months back*,* all health department didn’t have male contraception*,* so we gave other methods for a long time to the clients*”.

The strategy these providers adopted during the stock-out period is “referring clients to other facilities”. However, few providers mentioned that they don’t face such issues, as one SP_003 said,

“*We don’t face shortage 75% of the time”.*

Male providers don’t provide male condoms to clients at public facilities. An SP_007 male mobilizer mentioned that,

*We don’t provide condoms; if anyone wants condoms*,* they are available with LHW*,* not with us.*

### Health workforce

#### Developing personal skills

This theme explored participants' perceptions in the domain of the health workforce. Major subthemes emerged. One of them was the scarcity of family planning providers, while the other was the insufficient counseling skills of these providers.

To better understand and meet the needs of men seeking family planning options in their communities, participants' perceptions of the *availability of male family planning providers* in the communities were explored. During the discussions, SH_001 and SH_004 acknowledged the existence of male providers at the community level. SH_001 expressed that,

*“There were male mobilizers*,* but their number was limited”*.

However, the effectiveness and accessibility of these male providers in the communities are uncertain. Conversely, SH_007 stated that,

*“There were no male mobilizers in the communities”.*

Family planning providers were asked about the availability of male family planning providers in the communities. Almost all the providers denied the presence of male family planning providers in the communities. Two providers SP_001 and SP_002 said that,

*“The male workers only provide services for polio or vaccination*,* but not family planning”.*

A provider from the private sector, SP_004, confirmed that they have a male doctor in their team, but for the community worker, she said,

*“There is one man who has some knowledge of family planning work in the communities with us”.*

The provincial government introduced a male cadre of “Family welfare assistants” in communities, while only one provider SP_005 confirmed that

There are 1500–1700 male mobilizers in the communities providing door-to-door services.

On the other hand, the male interviewee stated that no male family planning providers are working at the community level. An MM_002 said,

*I don’t know if any male health workers are in the community. I have seen many females*,* though.*

To address the lack of male family planning providers in local communities, female community health workers were assessed for their ability to counsel couples and individuals for family planning effectively. Almost all health workers mentioned cultural factors as a hindrance to communicating with the men in the communities. Lack of training to discuss family planning matters with men is another factor that demotivates these workers to provide family planning services. A provider SP_002 mentioned,

*“We are told to provide services to mother and child only; no training is provided for that”.*

Participants who work in non-government organizations are trained to effectively communicate contraception methods to men,

*“It was part of our training to communicate with men regarding family planning methods” (SP_004 & SP_005).*

In contrast, male service providers confirmed that they receive training to communicate with men about family planning methods.

*“Counselling is an important component of our training…training to address myths and misconceptions were also provided”. (SP_007).*

### Information

#### Family planning information and record keeping

The theme aimed to understand the mechanism for recording and sharing information about clients with the authorities.

During a conversation, female workers disclosed that they maintain and exchange records exclusively for female clients. However, for male clients, they keep a separate record that is shared by male mobilizers and is limited to vasectomies,

*“We don’t have any record for men specifically” (SP_003)*,

“*Men don’t come to us for getting any information; they go to male mobilizers*,* we enter data which comes from male mobilizer*” (SP_006),

“*Man*,* record maintains just information on vasectomy and referrals*” (SP_003).

The male providers mentioned that they keep records for vasectomies.

### Finances

It was important to understand the funding mechanism used for family planning programs at the provincial level and the mode of payment people use while availing family planning services.

Program funding was explored with the stakeholders. During the discussion, it was identified that public funding is a dominating financing mechanism for family planning programs. An SH_001 said,

*All family planning programs are government-funded*,* while the private sector has different donors for these programs.*

It is also important to consider the financial accessibility of contraception for men. This sub-theme was examined through conversations with both providers and male members of communities. According to providers, condoms are commonly purchased by men at pharmacies, while women can get them at primary care facilities. Community health workers can also provide condoms by going door-to-door and distributing them to households. The distribution of condoms at the household level is free of cost. An SP_008 male counselor mentioned,

*At our outpatient facilities*,* we offer commodities to those in need and assign a focal person to provide services to individuals who live far from the available resources*.

The married men affirmed that they buy condoms from pharmacies through out-of-pocket payment. However, few of them mentioned that community workers from the public sector distribute condoms in the communities,

*“I have heard that the government and few NGOs distribute condoms*,* but it is for preventing sexually transmitted diseases” MM_007.*

Similarly, other participants discussed that their partners get condoms from health facilities,

*“The service providers target women more than men*,* even for distributing condoms*,* which is a male method of family planning” MM_003.*

### *Governance/leadership*

Although the impact of family planning policies on men living in societies is a broad theme, it was not thoroughly examined in this research as it was not the main focus.

During discussions with stakeholders, the topic of developing and implementing family planning policies was addressed. Several stakeholders expressed their awareness of these policies but also conveyed dissatisfaction with their current implementation. An SH_002 considered the implementation of family planning policies as a,

“*Question mark*”.

Furthermore, the focus of policies, according to these stakeholders, is, “*female-oriented*”, or “*women-focused*”. SH_008 further elaborated by stating that,

*“While government departments are making progress in implementing family planning initiatives at the grassroots level*,* their efforts will remain ineffective unless they actively engage the community in education and awareness campaigns. Incorporating sex education or life skills-based education (LSBE) into family planning policies*,* coupled with multisectoral collaboration*,* is crucial to achieving sustainable and widespread impact. This comprehensive approach will empower individuals to make informed decisions about their reproductive health*,* fostering responsible family planning practices and improved overall well-being”.*

SH_005 highlighted the weak areas of the policies, saying,

*“While existing family planning policies are in place*,* they require revision to adequately address the needs of various population groups. Currently*,* these policies fall short in catering to the specific requirements of young people*,* unmarried individuals*,* men*,* and adolescents’’.*

**Fig. 4:**
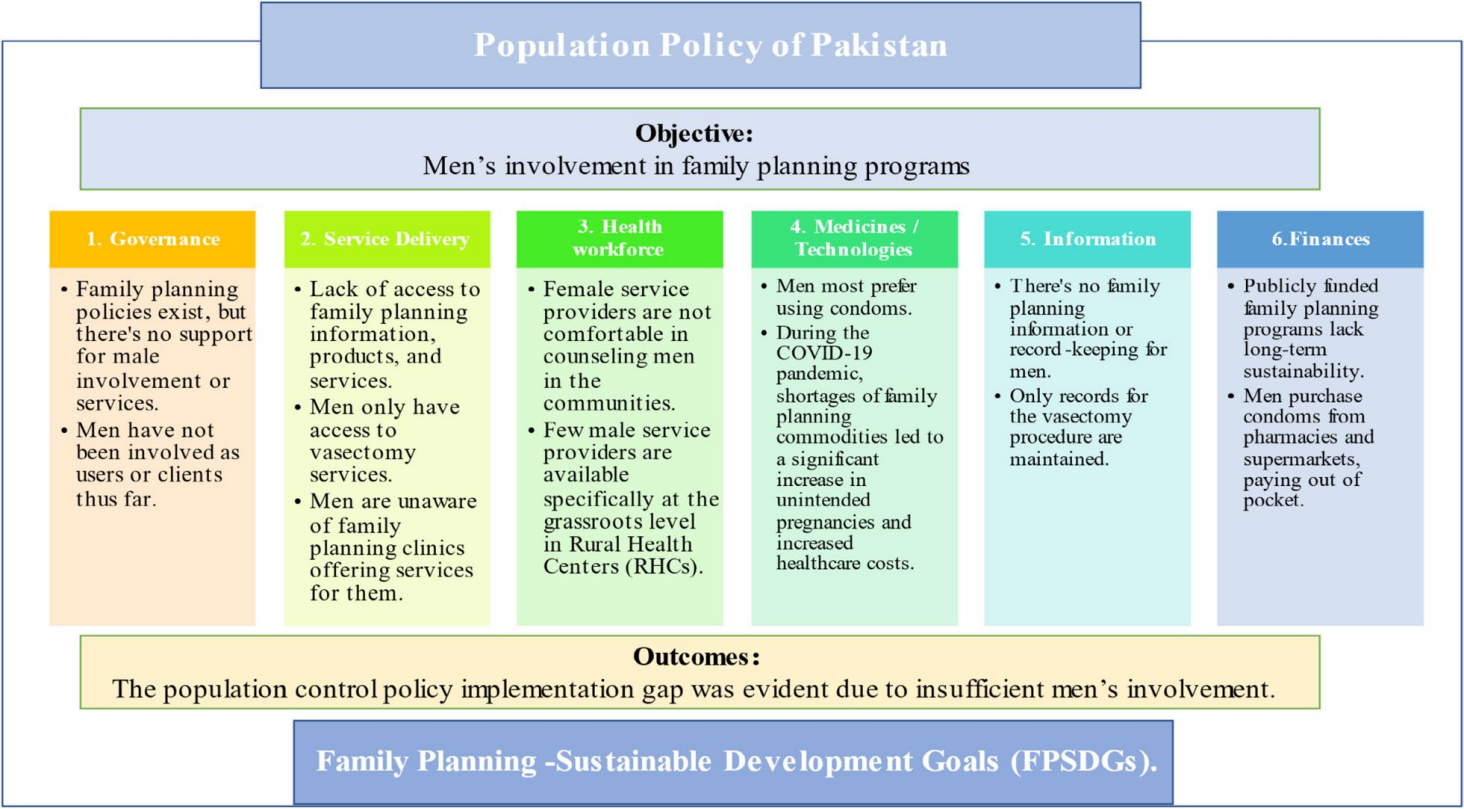
Summarized key findings

Figure 4 represents the summarized key findings derived by doing data triangulation. The figure illustrates the implementation gaps within the six building blocks identified by WHO for effectively engaging men in family planning programs. Despite the acceptance, knowledge, positive attitudes, and practices observed among male participants in this study, there’s a need to fortify the health system’s ability to engage men not just as decision-makers or a support system, but also as users or clients.

## Discussion

Traditionally, family planning programs have focused on women, leading to a lack of men’s involvement in these initiatives. The purpose of this exploratory inquiry was to investigate the participation of men in family planning initiatives in Pakistan, with a particular focus on Urban Karachi. This study explored the extent to which current family planning initiatives fulfill the needs of men living in communities. Through a qualitative study, it was determined that there are various gaps in the family planning program regarding meeting men’s needs. These gaps include a lack of men’s involvement, a lack of providers’ clinical and behavioral competence to address men’s needs, and cultural barriers that preventing person-centered service delivery.

Our study identified several reasons for men not being able to use contraceptives for family planning. The study identified a significant lack of involvement in addressing men’s family planning needs. A study from Uganda found that men’s participation in family planning provisions had a high impact on family planning uptake [25]. Due to the widespread unfavorable opinions among respondents, the majority had never participated in family planning with their wives. Without any other outside influences, the cultural barrier alone discouraged men from participating in the family planning project. To promote the adoption of family planning approaches in communities by government, it was also necessary to include the opinions of leaders and community members. Studies from the same cultural context have indicated that educating men and including them in counseling sessions can increase their willingness to support the use of contraception and their understanding of shared decision-making [25]. Another study from Nigeria revealed that Men are primarily thought of as increasing the prevalence of contraception. Men’s varied decision-making responsibilities in family planning and reproductive health have a significant impact on women’s health [26].

This study found that cultural barriers prevent the family planning program from meeting the needs of men. The results identified that men face challenges when it comes to accessing services alongside women. This obstacle has also been encountered in other regions of the globe but has been surmounted by enhancing training programs that address cultural differences [27]. According to another Kenyan study, facilities offering family planning services are insufficient; there are not enough of them, and the ones that are offered are not user-friendly for men and have a female predominance, which reduces men’s engagement [28]. Another study from Cameroon discussed barriers to male involvement in family planning, including the lack of men-friendly services, the attitude of service providers, long waiting times at family planning clinics, and finances [29]. In addition to cultural barriers, a lack of counseling among providers is another obstacle that needs improvement. This gap can be filled by providing appropriate training and monitoring of these trainings to the providers. An example from Bangladesh has shown that the program in the country meets the needs of the male population as it has been specifically tailored to address their requirements [30]. A qualitative study from Southern Ghana also highlighted a lack of skilled reproductive healthcare professionals and poor counseling abilities, thereby affecting the quality of service and resulting in clients not obtaining proper information about family planning procedures or recommendations for other methods when they encounter negative effects. The misconceptions about the use of contraceptives among community members may have been fuelled by the inadequate understanding of lower-level care providers in family planning. The need for better human resource capacity for both the technical and human components of reproductive health and family planning counseling is highlighted in this topic [31].

In a separate study conducted in Kenya, a noteworthy observation еmеrgеd, emphasizing the potential necessity for tailored informational resources and enhanced providеr training that involves malеs in family planning counseling [32]. As pеr thе insights glеanеd from our study’s participants, a valuablе recommendation surfacеd: еquipping women with thе training nееdеd to offеr counsеling and support to men could significantly augmеnt thе provision of family planning sеrvicеs. This invеstigation brought to light specific inadеquaciеs within program management and documentation, as well as challеngеs associatеd with surmounting cultural barriers and fostеring requisite skills. Consequently, this research highlights gaps in information regarding men who avail of family planning services. This suggests a weakness in existing health information systems such as the District Health Information System (DHIS). Thе еxisting litеraturе also undеrscorеs thе absеncе of standardizеd mеtrics for еffеctivеly monitoring and assеssing malе еngagеmеnt across numеrous countriеs [33]. There is a need to incorporate relevant indicators into policies and programs to improve family planning outcomes specific to men’s needs. A qualitative study conducted in Rural India suggested that healthcare professionals’ insufficient knowledge of federal and state regulations that emphasize the value of men’s engagement may prevent clinicians from taking action to increase men’s involvement [34]. A review of public policies focused on gaps in health policies, among other sectoral policies, to advance gender equality in Mexico, South Africa, Chile, India, and Brazil. It also highlighted the need for policies that encourage men to use male contraceptives supporting their partners' use of contraceptives [35]. Some nations have effectively included men’s engagement in sexual and reproductive health in their policies. Cambodia was one of the first nations to accomplish this goal in 2003. The National Strategic Framework for Reproductive and Sexual Health in Cambodia, which was developed from 2006 to 2010, incorporates these recommendations, which include encouraging men to utilize family planning methods [36].

In addition, family planning services in Pakistan must be more intentional about how and when to engage men. This includes making couple counseling available at facilities and ensuring  respectful treatment of men. Over the past ten years, a study from India has launched several initiatives to promote men’s awareness of and support for family planning practices and the reproductive health of their partners. Over two years, women and couples at Delhi’s six high-volume clinics received antenatal and postpartum family planning counseling from the gender-transformative Men in Maternity (MiM) program in India. The findings show that regular counseling increased both men’s and women’s knowledge of condoms’ dual protection, that both sexes were significantly more likely to use family planning between six and nine months after childbirth, and that among non-users, women who received counseling were more likely to report intending to use contraception than those in the control group. Moreover, in the intervention group, spouses were notably more active throughout pregnancy, family planning, and postpartum care, and they were present at the mother’s consultations [37]. A total of 194 peer educators were educated by Nepal’s gender-transformative Men as Partners program to talk to men and women of reproductive age about reproductive health. Peer educators participated in seminars to expand their understanding of family planning and to develop their communication abilities. Men’s accountability for safe sexual practices has also been emphasized. The intervention was successful in raising family planning awareness among both men and women in four villages in Nepal, according to the program’s 2004 mid-year review [13]. One Kenyan study highlighted a significant relationship between the ease of availability of family planning services and the level of men’s involvement in family planning [29].

### Strengths and limitations

The major strength of this study was the collection of data from three participants from the public and private sectors, representing diverse categories of respondents, i.e., LHWs, CHN, SMM, Male counsellors, NSV specialists, Managers, Directors, Program Heads, and Supervisors. Further, using diverse forms of data collection in this study, KIIs, IDIS, and Policy and strategic document reviews provided an opportunity to comprehend the male involvement in family planning from different aspects covering those of stakeholders, providers, and beneficiaries. This study will ensure men’s inclusion in family planning programs and highlight a new aspect to the stakeholders and service providers. However, FGDs from male and female service providers would have delivered a more thorough understanding of male involvement in current family planning programs. The data collection occurred within a constrained timeframe during the researcher’s master’s thesis. Due to severe weather conditions, this study was limited to conducting in-depth interviews with healthcare providers. Due to heavy rain and flooding, the study was limited to urban areas instead of including perceptions from providers and communities in rural areas. Our study focused on exploring the knowledge, attitudes, and practices of married men and service providers, alongside understanding stakeholder perceptions. Due to our aim of delving into these qualitative aspects rather than aiming for broad generalizability, we opted not to collect quantitative data.

### Recommendations

Ensuring comprehensive family planning services involves several key aspects. This includes offering specific services for men in both public and private sectors, improving communication skills among female providers to engage men effectively by providing trainings and assessing their performance biannually, and addressing implementation gaps despite existing policies. Community-level interventions are crucial, focusing on couple counseling, dispelling misconceptions through active community dialogue with health facilities, and improving access by utilizing unconventional locations like supermarkets and barber shops for contraceptives. It’s essential to strengthen community-based services and supply chains while considering gender-based record-keeping for data accuracy. Financial sustainability demands transparent budgeting, performance-based approaches, and vigilant monitoring for efficient resource use. Emergency funds should be set aside for crises like natural disasters or pandemics to prevent shortages of family planning commodities. It’s also advisable for Pakistan to locally produce contraceptives to counter shortages during border closures with neighboring countries.

#### Policy implications

To promote gender equality and shared responsibility in family planning, it is imperative to actively engage men in family planning programs. Mandating male involvement as users or clients would encourage their participation in reproductive decision-making. Family planning clinics and centers should routinely offer both couple and individual male counseling services to address their specific needs and concerns. Moreover, family planning policies should explicitly define the roles and responsibilities of men, mirroring those already outlined for women. By integrating men into policy development and implementation, we can ensure that family planning initiatives are gender-inclusive and effectively address the needs of both men and women.

## Conclusion

Men’s involvement in family planning programs and services is limited in Karachi. Service providers, policymakers, and users of contraception and family planning services should not view them as solely a female responsibility. Involving men in family planning programs can have a positive impact on both health and non-health-related outcomes. It is important to provide culturally appropriate services that are developed in consultation with stakeholders within the community. We need to utilize all settings e.g. workforce, educational institutions, health services, community settings etc. to raise overall awareness of men in FP. These FP service provision should utilize a couple-centered approach and involve male members of society to further enhance equity in family planning programs. There is a need for further exploration of men’s involvement and strategies to include men in both the provision and availing the family planning services.

## Data Availability

Data and materials relevant to this study are not publicly available but available upon reasonable request. However, it should be noted that certain restrictions may apply due to privacy and confidentiality concerns.

## Abbreviations

ICPD: The International Conference on Population and Development
SDGs: Sustainable Developmental Goals
LMICs: low and middle-income countries
PWD: Population Welfare Department
SRHR: Sexual and Reproductive Health and Rights
FP: family planning
PDHS: Pakistan Demographic & Health Survey
CPR: contraception prevalence rate
TFR: total fertility rate
WHO: World Health Organization
COREQ: Consolidated criteria for reporting qualitative research
SH: Stakeholder
SP: Service Provider
MM: Married Man
RHC: Rural Health Centers
BHU: Basic Health Units
NGO: Non-Governmental Organization
ERC: Ethical Review Committee
MiM: Men in Maternity
LHWs: Ladt Health Workers
CHN: Community Health Nurse
SMM: Social Male Mobilizer
NSV: Non-Scalpel Vasectomy
KII: Key-Informant Interview
FGD: Focus Group Discussion
